# Machine Tool Wear Prediction Technology Based on Multi-Sensor Information Fusion

**DOI:** 10.3390/s24082652

**Published:** 2024-04-21

**Authors:** Kang Wang, Aimin Wang, Long Wu, Guangjun Xie

**Affiliations:** 1 Digital Manufacturing Institute, Beijing Institute of Technology, Beijing 100081, China; 3120195216@bit.edu.cn; 2 School of Mechanical and Electrical Engineering, Shandong Jianzhu University, Jinan 250101, China; wulong@sdjzu.edu.cn; 3 Department of Mechanical Engineering, Nanjing University of Science and Technology, Nanjing 210094, China; 121101221938@njust.edu.cn

**Keywords:** tool wear prediction, LSTM network, deep residual network, multi-sensor information fusion

## Abstract

The intelligent monitoring of cutting tools used in the manufacturing industry is steadily becoming more convenient. To accurately predict the state of tools and tool breakages, this study proposes a tool wear prediction technique based on multi-sensor information fusion. First, the vibrational, current, and cutting force signals transmitted during the machining process were collected, and the features were extracted. Next, the Kalman filtering algorithm was used for feature fusion, and a predictive model for tool wear was constructed by combining the ResNet and long short-term memory (LSTM) models (called ResNet-LSTM). Experimental data for thin-walled parts obtained under various machining conditions were utilized to monitor the changes in tool conditions. A comparison between the ResNet and LSTM tool wear prediction models indicated that the proposed ResNet-LSTM model significantly improved the prediction accuracy compared to the individual LSTM and ResNet models. Moreover, ResNet-LSTM exhibited adaptive noise reduction capabilities at the front end of the network for signal feature extraction, thereby enhancing the signal feature extraction capability. The ResNet-LSTM model yielded an average prediction error of 0.0085 mm and a tool wear prediction accuracy of 98.25%. These results validate the feasibility of the tool wear prediction method proposed in this study.

## 1. Introduction

With the rapid development of modern industries and scientific technology, manufacturing equipment is gradually becoming larger, more integrated, faster, more automated, and intelligent. In the manufacturing industry, computer numerical control (CNC) milling is widely used, and the importance of cutting tools is evidenced by how they directly affect the dimensional accuracy and surface quality of products. In addition, it is more convenient to replace tools according to the specific piece and time required to cope with large-scale processing environments. However, this method has certain limitations. First, it relies heavily on worker experience to judge tool wear. Second, replacing tools through piece and time methods cannot accurately determine the service life of the tools, which may lead to unnecessary tool waste and, more significantly, affect the quality of the products. Developing tool wear prediction technology can avoid tool damage and other problems, as well as helping to improve tool chip speed and leading to substantial savings in production costs [1].

Tool wear prediction methods can be divided into two general categories: direct and indirect [2]. Direct measurement methods involve directly measuring tool wear using equipment such as microscopes to determine the degree of tool wear. In contrast, indirect measurement methods predict tool wear based on relevant machining parameters. Using high-magnification microscopes to directly capture images of the cutting edges of tools can yield more accurate measurement results. However, this method also has limitations. Because machine tools cannot be arbitrarily stopped during the machining process, they cannot be dismantled and measured at any time. Therefore, indirect measurement methods have been extensively investigated.

Some researchers have used a single sensor to collect information that can characterize tool wear. For example, Xu et al. [3] conducted experiments on high-speed steel drills using the wavelet packet transform coefficients of the cutting force and torque as inputs to train a backpropagation (BP) neural network model. In addition, Liu and Kumagai [4] developed a system for monitoring drill wear during boring processes using a combination of BP neural networks and adaptive fuzzy reasoning to monitor tool wear classification, achieving a wear classification accuracy of 100%. In addition to traditional indirect monitoring methods that use current, power, vibration, and force sensors, scholars have also explored various other types of sensor signals for monitoring tool wear in CNC machine tools (e.g., tool temperatures [5] exhibited in the machining process) and have conducted tool stress analyses [6]. The exploration and applications of these methods provide diverse options for tool wear monitoring, enabling research on CNC machine tool processing technology to be more comprehensive and diverse.

Many studies on multi-sensor fusion have been conducted. For example, Kul et al. [7] developed a multi-sensor flutter detection system for industrial sites, collecting data using accelerometers and axial force sensors, and used neural network technology with wavelet packet decomposition processing and analysis techniques for flutter monitoring. This method, which combined multiple sensors and advanced signal processing techniques, aimed to improve the accuracy and reliability of flutter monitoring by providing effective real-time monitoring and warnings regarding the status of industrial equipment. In addition, Othman et al. [8] comprehensively analyzed different vibration signals and methods for processing acoustic emission signals and compared the diagnostic results of the fused signals with those of single-signal sources, demonstrating the superiority of fused signals. Furthermore, Duro [9] constructed a multi-sensor fusion framework for monitoring the machining process of CNC machine tools, combining all the key steps from signal feature extraction, data filtering, data normalization, and standardization for weight allocation and data fusion. Using a combination of maximum likelihood estimation and autocorrelation coefficient analysis, signals from different mounting positions of acoustic emission sensors were fused together. Moreover, Segreto [10] built a BP neural network to improve the tool wear prediction accuracy by collecting and fusing cutting force, acoustic emission, and vibration signals. These examples highlight the widespread application of information fusion technology in different fields to improve the accuracy and performance of tool monitoring and diagnosis and optimize the results of the decisions required to maintain tools. Bagga et al. [11] proposed a multi-sensor data fusion method to measure and predict rear tool wear using various parameters, such as vibration, power, temperature, force, and surface roughness, and constructed an artificial neural network model for tool wear measurement and prediction. Wang et al. [12] proposed a novel virtual tool wear sensing technology based on multi-sensor data fusion and artificial intelligence models, fusing multi-sensor data (such as force and vibration signals) with dimensionality reduction techniques and support vector regression models to infer tool wear parameters that are difficult to measure.

Generally, tool wear states are predicted by extracting feature signals from detection signals. Traditional machine learning methods are widely used in the fault diagnosis of mechanical equipment and tool wear prediction. For example, Zhu [13] and Jia et al. [14] used sparse decomposition theory and autoencoder neural network technology to diagnose mechanical equipment faults, achieving superior prediction results. In addition, He et al. [15] proposed the construction of a dynamic Bayesian network model and used fused data as monitoring signals to predict tool wear. Furthermore, Cheng et al. [16] proposed a method combining empirical mode decomposition to extract latent features, and constructed a tool wear prediction model based on generalized multiclass support vector machines. These studies demonstrate the diverse applications of traditional machine learning methods in tool wear prediction.

Deep learning methods are also very common in the fields of feature extraction and tool wear prediction. Convolutional neural networks (CNNs) are typically used to extract key features and predict tool wear amounts [17]. For example, Lu et al. [18] proposed the use of shallow CNNs in the feature extraction of monitoring signals. In addition, Kong et al. [19] proposed a tool wear prediction model based on an integrated radial basis function kernel principal component analysis (KPCA_IRBF) and a relevant vector machine (RVM). Compared with traditional methods, such as partial least squares (PLS), artificial neural networks (ANN), and support vector machines (SVM), the RVM method provided more accurate predictions and offered additional advantages in terms of confidence intervals. Zhang et al. [20] proposed an improved integrated estimation method based on long short-term memory (LSTM) networks and particle filter (PF) algorithms. The integrated PF-LSTM recognition method predicted the random tool wear process based on historical measurement data, and the accuracy of the PF-LSTM method was verified through micromilling experiments.

Mathematical and modeling methods are also used to diagnose tool wear. For example, Awasthi et al. [21] developed a physics-based digital twin method for tool wear diagnosis during machining. For milling tools, information theory methods were used to optimize the test design and sensor suites were used for fault detection, thereby improving the inference of the tool wear. The robustness of the design was verified using dynamic time warping and k-NN classification methods. Li et al. [22] proposed a new physics-based meta-learning framework to predict tool wear at different wear rates. Piecewise fitting parameters were used to combine data-driven analysis and parameter estimation, which ensured the accuracy of the parameters, improved the interpretability of the tool wear prediction, and accurately reflected changes in tool wear rates.

However, regardless of whether a single-sensor detection method, multi-sensor fusion method, or machine learning algorithm is used, none of these methods consider the influence of multiple operating conditions during processing. Most methods primarily focus on monitoring processing under a single working condition and cannot adapt to the complex and dynamic conditions in actual processing situations.

Therefore, in this study, we conducted an analysis of the characteristics of the processed parts to select appropriate sensors as signal sources. To ensure the processing quality and efficiency of the parts and avoid losses caused by tool breakage, a tool wear prediction technology based on multi-sensor information fusion is proposed. The technology monitors changes in tool status during the processing of thin-walled parts. To improve the accuracy of tool wear prediction, data collected by sensors during processing were used for model training and prediction, and a predictive model for tool wear based on combining the ResNet and long short-term memory (LSTM) models (called ResNet-LSTM) was constructed. Experimental data for thin-walled parts under various machining conditions were utilized to monitor the changes in tool conditions. The proposed ResNet-LSTM model significantly improved the prediction accuracy compared to the individual LSTM and ResNet models.

**Figure 1:**
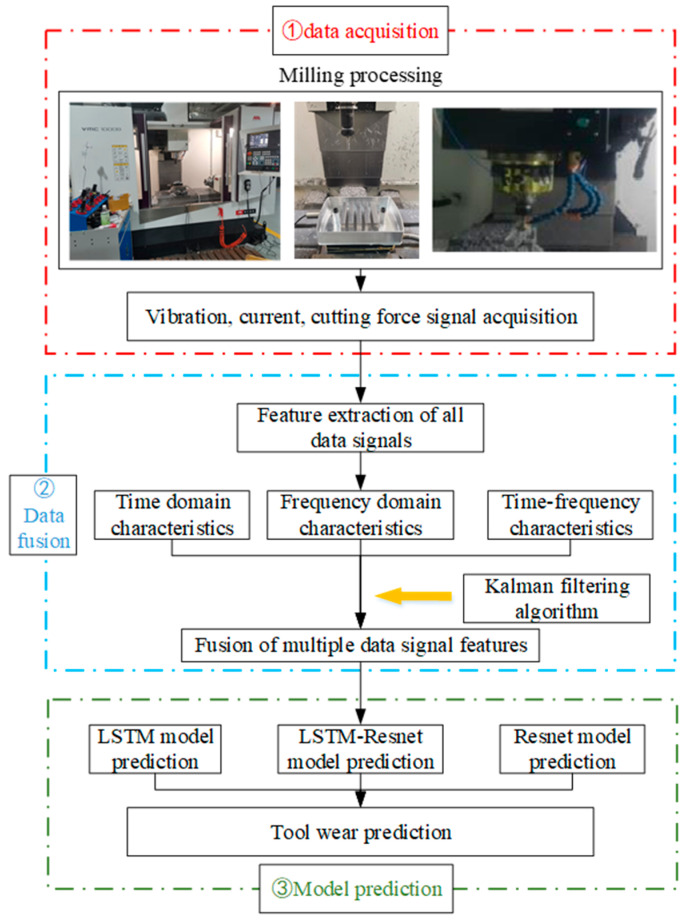
Diagram of the basic structure of the method developed in this study.

The basic structure of the method developed in this study is shown in Figure 1. The rest of this paper is organized as follows. Section 2 introduces the data fusion method and describes the construction of the model, and Section 3 describes the data collection process. Section 4 analyzes the results of the processing experiments and model predictions, and validates the accuracy of the model. Finally, Section 5 concludes the study.

## 2. Data Fusion Method and Model Construction

### 2.1. Multi-Sensor Information Fusion Technology

The complexity of the tool-cutting process results in the generation of signals in a non-stationary state, which poses challenges for tool monitoring. Traditional single-sensor monitoring methods can reduce the accuracy and reliability of analyses, particularly when they are utilized improperly. In addition, the complex and interrelated structures of machine tool systems can easily lead to one-sidedness when single-sensor monitoring methods are used.

Multi-sensor information fusion technology is a comprehensive automated information-processing method that has become widely researched. Bayesian inference, Kalman filtering, fuzzy set theory, neural networks, and wavelet analysis methods are commonly used for information fusion. The application of these methods enables more accurate data processing and more effective decisions, thereby improving the performance and reliability of systems. The main goal of information fusion is to extract as much valid information as possible from the measured objects and environment by optimizing the combination of observations from various sensors.

**Figure 2:**
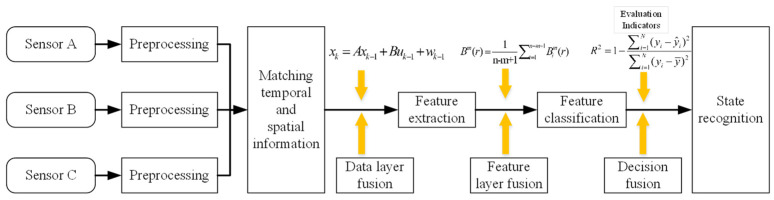
Structure of a state-recognition system based on multi-sensor information fusion.

The structure of a state-recognition system based on multi-sensor information fusion is illustrated in Figure 2.

This study utilized the weighted observation fusion Kalman estimation algorithm to handle the problem of fusing large amounts of data from multiple sensors. Details of the equations can be found in Reference [23]. Based on data fusion, the initial values of $x_{0}$ and $P_{0}$ are set. At time *k*, measurements are obtained from the sensors, and these values are denoted as *z*. Then, using a recursive method, the state estimation value at time *k*, denoted as $x_{k}(k=1,2,\cdot \cdot \cdot ,N)$, is calculated. These steps are repeated continuously until the estimation requirements are satisfied, which terminates the recursive calculations. The basic principle of Kalman filtering involves the “predict-measure-correct” logical sequence to eliminate interference data from the collected sensor data and reconstruct the system’s state vector using the measured values, thereby effectively estimating the state data. The state equation of the system infers the current state based on the previous state and control variables, and is calculated as follows:

(1) $$x_{k}=Ax_{k-1}+Bu_{k-1}+w_{k-1},$$

where $x_{k}$ is the *n*-dimensional vector of state components, *A* denotes the state transition matrix, $u_{k-1}$ is the external input that the system can accept, *B* is the matrix that converts the inputs into states, and $w_{k-1}$ is the noise of the prediction process (corresponding to the noise of each component in $x_{k}$), with an expectation of 0 and a covariance of *Q,* representing Gaussian white noise. The system’s observation equation is expressed as follows:

(2) $$z_{k}=Hx_{k}+v_{k},$$

where $z_{k}$ is the measurement value and input of the filter, *H* is the matrix used to transform the state variables, and $v_{k}$ is the observation noise that follows a Gaussian distribution *N*(0, *R*). The basic steps involved in the Kalman filter are as follows:

Step 1: Predict an estimate:

(3) $${\hat{x}}_{\bar{k}}=A{\hat{x}}_{k-1}+Bu_{k-1}.$$

Step 2: Compute the covariance:

(4) $$P_{\bar{k}}=AP_{k-1}A^{T}+Q.$$

Step 3: Compute the Kalman gain $K_{k}$:

(5) $$K_{k}=\frac{P_{\bar{k}}H^{T}}{HP_{\bar{k}}H^{T}+R}.$$

The noise *w* (system error) and observation noise *v* (measurement error) in the state and measurement equations are generally assumed to be Gaussian white noise that follows a normal distribution *P*(*w*)–(0, *Q*), *P*(*v*)–(0, *R*), where *Q* and *R* are different covariance matrices at time *k*:

(6) $$Q=E[w_{k},w_{k}^{T}],R=E[v_{k},v_{k}^{T}].$$

Step 4: Update the estimate:

(7) $${\hat{x}}_{k}={\hat{x}}_{\bar{k}}+K_{k}(z_{k}-H{\hat{x}}_{\bar{k}}).$$

Step 5: Update the estimate covariance for the next time step using the following:

(8) $$P_{k}=(I-K_{k}H)P_{\bar{k}},$$

where ${\hat{x}}_{k}$ and ${\hat{x}}_{k-1}$ represent the posterior state estimate values at times *k* and *k* − 1, respectively (which is one of the results of the filtering process); ${\hat{x}}_{\bar{k}}$ is the prior state estimate value at time *k* (which is an intermediate calculation of the filtering); $P_{k}$ and $P_{k-1}$ represent the posterior estimate covariance at times *k* and *k* − 1, respectively (which are one of the results of the filtering process); and $P_{\bar{k}}$ is the prior estimate covariance at time *k* (which is an intermediate calculation of the filtering).

In this study, the spindle current, cutting force, and vibration signals were detected by sensors during the CNC machining process because they have the greatest influence on the state of the tool and can best characterize its state. These signals were then used for tool wear monitoring via multi-sensor signal fusion.

### 2.2. Signal Denoising and Feature Extraction Methodology

#### 2.2.1. Wavelet Packet Transform

In the practical collection of spindle vibration signals from machine tools, the obtained signals often contain not only the original vibration signal, but also other noise or interference signals with high randomness. Noise signals are a common problem in signal analysis and may originate from various sources of interference, such as electromagnetic waves and mechanical vibrations. In the analysis process, a series of denoising measures is required to reduce the influence of noise, thereby improving the reliability and accuracy of the signal.

The wavelet packet transform is a multiscale time-frequency domain transformation method commonly used in signal analysis [24]. It can decompose high-frequency band signals into subsignals with local characteristics, thereby providing more detailed information about the signal. This method can be applied to analyze and extract changes in the state of the monitoring equipment [25].

**Figure 3:**
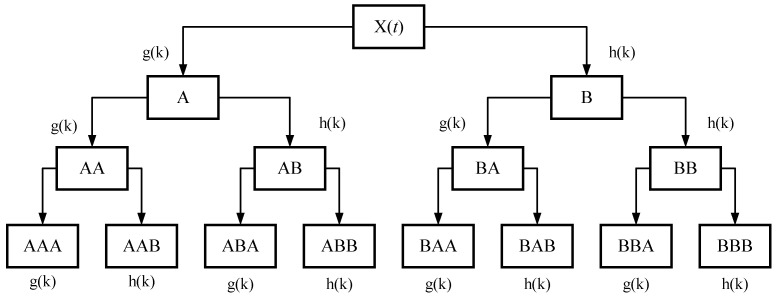
Tree structure of the three-level wavelet packet decomposition.

The wavelet packet is defined as follows:

(9) $$\left\{ \begin{array}{l} u_{2n}^{j}(t)=\sqrt{2}\sum_{k}h(k)u_{n}^{j}(2t-k)\\ u_{2n+1}^{j}(t)=\sqrt{2}\sum_{k}g(k)u_{n}^{j}(2t-k) \end{array}\right.(n=0,1,2,\cdot \cdot \cdot ;k=0,1,2,\cdot \cdot \cdot m).$$

when decomposing using a low-pass filter, the coefficients are denoted as *h*(*k*); for a high-pass filter, the coefficients are denoted as *g*(*k*). At the *j*-th level of the wavelet packet decomposition, there are a total of $2^{j}$ wavelet packet bases, denoted as *j*. When *n* = 0, the scaling function ϕ(t) and basic wavelet function ψ(t) are defined as follows:

(10) $$\left\{ \begin{array}{l} u_{0}^{0}=\phi (t)\\ u_{1}^{0}=\psi (t) \end{array}\right.,$$

respectively. Using the method for determining the number of decomposition levels mentioned above, the optimal number of decomposition levels was determined to be three. Therefore, the signal was subjected to three-level wavelet packet decomposition, as shown in Figure 3.

In the figure, signal *X*(*t*) represents the original signal before decomposition. This is decomposed into a low-frequency component signal (obtained using low-pass filter coefficients *g*(*k*)) and a high-frequency component signal (obtained using high-pass filter coefficients *h*(*k*)). The high- and low-pass filter coefficients must satisfy the following orthogonal relationship:

(11) $$g(k)={\left(-1\right)}^{k}h(1-k).$$

The decomposed signals obtained at different decomposition levels are calculated layer-by-layer using the following equations:

(12) $$s_{i+1,2j}(n)=\sum_{k}g(k-2n)s_{i,j}(k),$$

(13) $$s_{i+1,2j+1}(n)=\sum_{k}g(k-2n)s_{i,j}(k).$$

Following the aforementioned decomposition method, after the signal undergoes wavelet packet decomposition at the *i*-th level, $2^{i}$ characteristic signals are obtained, each corresponding to a specific frequency band.

#### 2.2.2. Time-Frequency Domain Feature Extraction Based on Wavelet Packet and Sample Entropy

Sample entropy, proposed by Richman and Moorman in 2000 as an improvement on approximate entropy, is a method for measuring the complexity of a time series. This method can be used to analyze the time series obtained from continuously sampled processes. In theory, the sample entropy reflects the irregularity and complexity of signals and is considered a useful tool for analyzing vibration signals [26]. By applying sample entropy, a better understanding of the characteristics of the vibration signals can be attained. The specific steps of the algorithm are as follows.

Step 1: Assume that the sampling obtains an *n*-dimensional time series x(1),x(2),…,x(n) with equal time intervals.

Step 2: Denoting the pattern dimension as *m*, construct an *m*-dimensional vector from the original sequence:

(14) x(i)=[x(i),x(i+1),⋅⋅⋅,x(i+m−1)],i=1,2,⋅⋅⋅,n−m+1.

Step 3: Define the distance between *x*(*i*) and *x*(*j*) as follows:

(15) $$d(i,j)=\underset{k=1∼m-1}{max}\left|x(i+k)-x(j+k)\right|,k=0,1,\cdot \cdot \cdot ,m-1.$$

Step 4: Set a threshold value *r*, and for each *i*, compute the ratio of the number of d(i,j)<r occurrences to the distance *n – m + 1*, denoted as $B_{i}^{m}(r)$:

(16) $$B_{i}^{m}(r)=\frac{\mathrm{COUNTIFS}[d(i,j)<k]}{n-m+1},1\leq j\leq n-m,i\neq j.$$

Calculate the mean of $B_{i}^{m}(r)$ for all *i* values:

(17) $$B^{m}(r)=\frac{1}{n-m+1}B_{i}^{m}(r).$$

Step 5: For *m +* 1 dimensions, repeat steps (2)–(4) to obtain $B_{i}^{m+1}(r)$. The sample entropy of the sequence is then obtained as follows:

(18) $$SampleEn(m,r)=\underset{n\to \infty }{\lim}\left[-\ln\frac{B^{m+1}(r)}{B^{m}(r)}\right].$$

In practical vibration signals, *n* adopts a finite value; therefore, the estimated sample entropy of the sequence is:

(19) $$B^{m}(r)=\frac{1}{n-m+1}\sum_{i=1}^{n-m+1}B_{i}^{m}(r).$$

### 2.3. LSTM-Based Tool Wear Prediction Model

LSTM is a special variant of recurrent neural networks (RNN). It features unique “gate” structures that address the drawbacks of traditional RNNs, such as the problem of weight impacts being too significant (which leads to issues such as gradient explosion or vanishing). LSTM networks converge faster and more effectively, resulting in an improved prediction accuracy.

**Figure 4:**
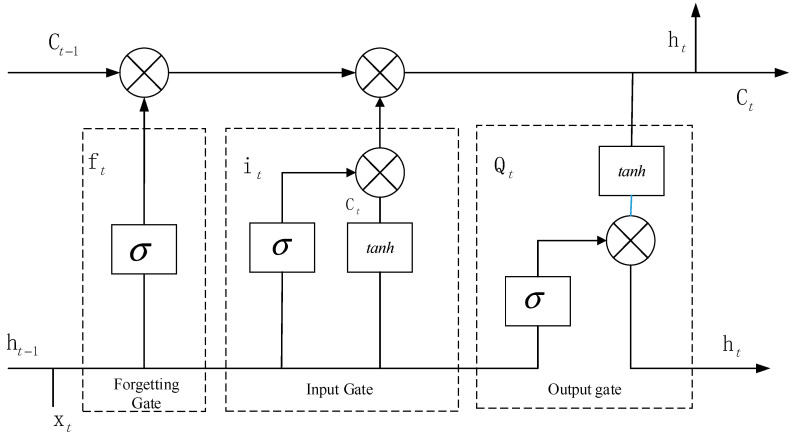
Diagram of the basic structure of LSTM.

LSTM networks consist of three crucial gates: forget, input, and output. These gates collaborate to determine what information is memorized and forgotten at each moment. Specifically, at each moment, they control the amount of new information added to the cell, whether information is forgotten, and whether any information is used as output. This gate control mechanism enables LSTM networks to more effectively capture long-term dependencies in time-series data, qualifying them as excellent tools for processing data with temporal properties, such as speech and text. In addition, the gate mechanisms of LSTM effectively address the issues with traditional RNNs, making neural networks more suitable for handling sequential data as well as improving model performance and learning capabilities. The basic structure of LSTM is illustrated in Figure 4.

Equations are detailed in Reference [27]. In the forget gate, a sigmoid function determines the information discarded from the cell state and is expressed as follows:

(20) $$\Gamma_{f}=\sigma (\omega_{f}\left[\alpha^{t-1},x^{t}\right]+b_{f}),$$

where the output at time step *t* − 1 is denoted by $\alpha^{t-1}$, the input at time *t* is denoted by $\alpha^{t-1}$, the weight of each variable is represented by $\omega_{f}$, the bias term is denoted by $b_{f}$, and σ(x) represents the form of the sigmoid function, which is defined as follows:

(21) $$\sigma (x)={\left(1+e^{-x}\right)}^{-1},$$

where $\Gamma_{f}$ ranges between 0 and 1, which indicates the extent to which each value in the cell state $c^{t-1}$ should be preserved; a value of 1 indicates “fully retained” and a value of 0 indicates “completely discarded”.

Updating the information stored in the cell state is the primary function of the output gate and involves the following three steps.

Step 1: The sigmoid function of the input gate is used to compute the result $\Gamma_{u}$, which determines which values to update.

Step 2: A new candidate value vector ${\tilde{c}}^{\left(t\right)}$ is created based on the tanh function and added to the new cell.

Step 3: The old cell state is multiplied by the forget gate to forget some of the old information. Then, the product of $\Gamma_{u}*{\tilde{c}}^{\left(t\right)}$ is added. The new candidate value continuously changes the degree of each state. Finally, the current cell state is updated. The formulas are expressed as follows:

(22) $$\Gamma_{u}=\sigma (\omega_{u}\left[\alpha^{t-1},x^{t}\right]+b_{u}),$$

(23) $${\tilde{c}}^{\left(t\right)}=tanh(\omega_{c}\left[\alpha^{t-1},x^{t}\right]+b_{c}),$$

(24) $$c^{t}=\Gamma_{u}*{\tilde{c}}^{\left(t\right)}+\Gamma_{f}*c^{t-1}.$$

The $\Gamma_{u}$ values range from 0 to 1, whereas the tanh function is a hyperbolic tangent activation function with an output range of −1–1. Therefore, the cell state value at time *t* − 1 is denoted as $c^{t-1}$, ${\tilde{c}}^{\left(t\right)}$ and represents the recorded information to be extracted from the input information at time *t*, while $c^{t}$ denotes the updated cell state value. 

The sigmoid function determines the amount of output information controlled by the output gate. The value of $c^{t}$ is determined using the tanh function to obtain the output value at time *t*. This can be achieved by multiplying $\Gamma_{0}$ and $c^{t}$, as expressed by

(25) $$\Gamma_{0}=\sigma (\omega_{0}\left[\alpha^{t-1},x^{t}\right]+b_{0}),$$

(26) $$\alpha^{t}=\Gamma_{0}*c^{t}.$$

Finally, processing within a single neuron requires the assistance of three control gates, a mechanism that allows the highest utilization of input data, and the formation of memories of past long-term data in the LSTM model.

(1) Building the LSTM network model

**Figure 5:**
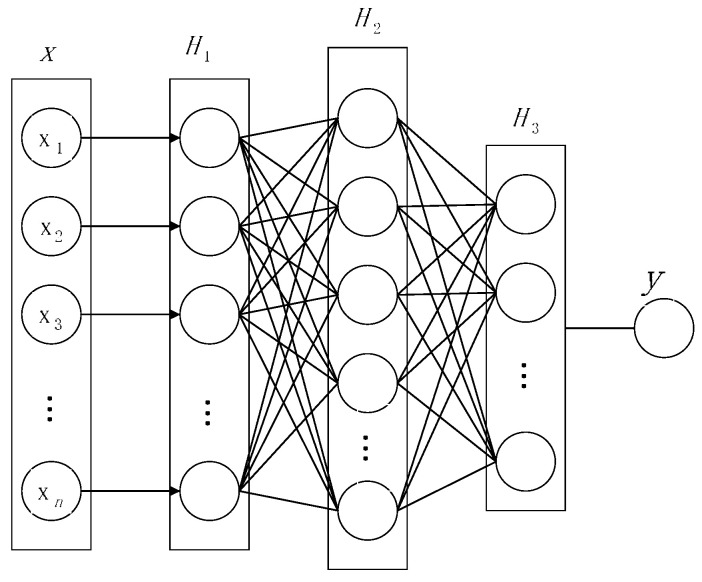
Structure of the LSTM network model.

According to [27], the use of up to three layers yields optimal results for LSTM models. Therefore, a two-layer LSTM network was constructed for this experiment. Its structure is displayed in Figure 5.

First, the data collected from the sensors, including the X-, Y-, and Z-axis vibrations, force signals, and the current signal, were preprocessed. When each signal component was treated separately, the input layer dimension was set to 6, resulting in $X=[x_{1},x_{2},x_{3},x_{4},x_{5},x_{6},x_{7}]$. However, when the feature vectors were used as the input, the input layer dimension was set to 40, resulting in $X=[x_{1},x_{2},x_{3},\ldots ,x_{40}]$.

Next, the number of neurons in the hidden layer was set to 100 to retain both the long- and short-term memory information. Subsequently, the number of neurons in the hidden layer was adjusted to 50 and then reduced to 20 before proceeding with tool wear prediction. The dimension of the fully connected output layer was set to 1, enabling the tool wear to be predicted based on the output value. This structural design aimed to fully utilize the hierarchical structure of neural networks and memory units at different levels to achieve a more accurate tool wear prediction.

(2) Network parameter configuration

Step 1: Normalization:

The data were normalized via

(27) $$x^{\prime }=\frac{x-min(x)}{max(x)-min(x)}.$$

Step 2: Loss function calculation:

The root mean square error (RMSE) was selected as the loss function in the LSTM prediction; it was defined as follows:

(28) $$RMSE=\sqrt{\frac{1}{T}\sum_{t=1}^{T}{\left(y_{t}-{\bar{y}}_{t}\right)}^{2}},$$

where $y_{t}$ represents the predicted value, ${\bar{y}}_{t}$ is the true value, and *T* is the number of samples.

Step 3: Evaluation metrics:

The selection of the evaluation metrics significantly affected the assessment of the experimental results. In this study, three coefficients, namely, the mean absolute error (MAE), RMSE, and coefficient of determination $R^{2}$, were chosen as indicators to evaluate the model’s prediction capability. The latter ($R^{2}$) represents the degree of fit between the predicted and actual data (the higher the value of $R^{2}$, the better the fit), and served as the criterion to determine the accuracy of the model’s predictions. It is expressed as follows:

(29) $$R^{2}=1-\frac{\sum_{i=1}^{N}{\left(y_{i}-{\hat{y}}_{i}\right)}^{2}}{\sum_{i=1}^{N}{\left(y_{i}-\bar{y}\right)}^{2}},$$

where $y_{i}$ represents the true value, ${\hat{y}}_{i}$ denotes the predicted value, and $\bar{y}$ is the mean of the actual values. The initialization parameters for the LSTM network model are shown in Table 1.

### 2.4. Predictive Model of Tool Wear Based on ResNet

**Figure 6:**
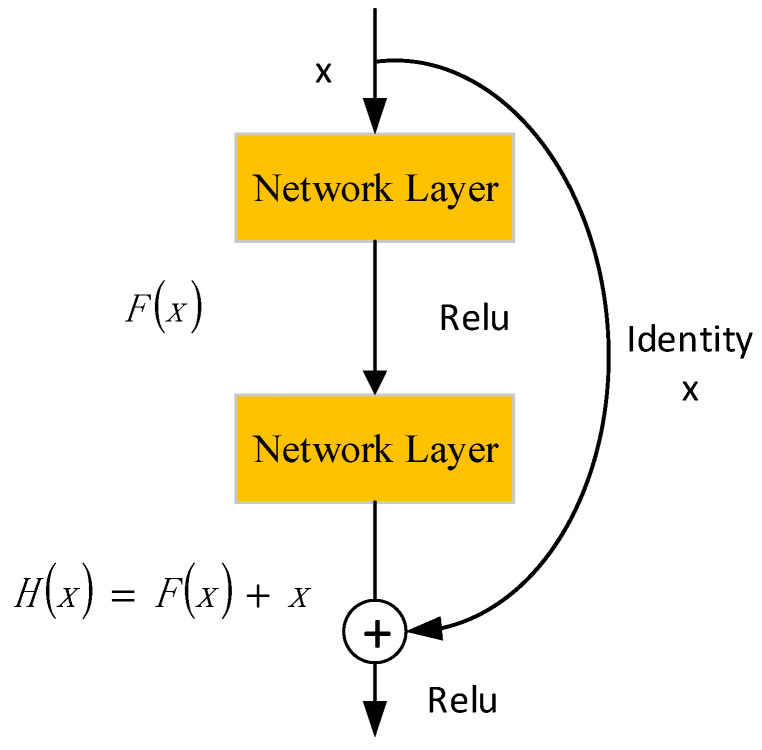
Structure of residual modules.

ResNet addresses an insufficiency in feature extraction capability by introducing the concept of identity mapping. This concept allows the network to learn residuals instead of directly learning low-level features, thereby facilitating gradient propagation. The ResNet network model proposed by Yu et al. [28] consists of multiple residual modules that are stacked together. The structure of these residual modules helps maintain a stable gradient propagation, enabling the network to learn features at deeper levels. The structure of residual modules is shown in Figure 6.

Firstly, the residual module transforms the input *x* into an output *H*(*x*). Here, *H*(*x*) can be computed by simply adding *F*(*x*) and *x: H*(*x*) = *F*(*x*) + *x*. This formula indicates that the output *H*(*x*) is composed of the residual part *F*(*x*) and the input *x*. The purpose of this design is to maintain the integrity of information propagation via identity mapping, which maps the input directly to the output without any change. By introducing identity mapping, residual networks can prevent the degradation of network performance as the depth increases.

Second, networks designed with identity mapping can focus on learning the residual part *F*(*x*). Because the identity mapping part remains unchanged, the network only needs to focus on learning how to better utilize the residual information to improve performance. The advantage of this design is that it simplifies the complexity of the training process. Researchers can focus more on optimizing the residual part to enhance the network’s learning ability without being concerned with how identity mapping will degrade the performance. This approach significantly reduces the difficulty of network training because the model only needs to capture the differences between the input and expected outputs.

In the predictive model, metrics such as the MAE, RMSE, and $R^{2}$ were primarily used as evaluation indicators, and they were defined as follows:

(30) $$MAE=\frac{\sum_{i=1}^{n}\left|y_{i}-{\hat{y}}_{i}\right|}{n},$$

(31) $$RMSE=\sqrt{\frac{\sum_{i=1}^{n}{\left(y_{i}-{\hat{y}}_{i}\right)}^{2}}{n}},$$

(32) $$SSR=\sum_{i=1}^{n}{\left({\hat{y}}_{i}-\bar{y}\right)}^{2},$$

(33) $$SSE=\sum_{i=1}^{n}{\left(y_{i}-{\hat{y}}_{i}\right)}^{2},$$

(34) $$SST=SSR+SSE=\sum_{i=1}^{n}{\left(y_{i}-\bar{y}\right)}^{2},$$

(35) $$R^{2}=1-\frac{SSE}{SST},$$

where $y_{i}$ represents the true tool wear value, ${\hat{y}}_{i}$ represents the tool wear value predicted by the model, $\bar{y}$ represents the mean of the predicted values, *SSR* is the “sum of squares due to regression” and measures the total variation explained by the regression model, *SSE* is the “sum of squares due to error” and measures the variation that is unexplained by the regression model, and *SST* is the “total sum of squares” and represents the total variation in the true tool wear values. In regression analysis, these metrics are fundamental for assessing how well the model’s predictions align with the actual values and how much of the total variation in the data is explained by the model.

## 3. Data Collection Experiment

### 3.1. Introduction to Tool Wear States

Tool wear can be broadly categorized as normal or abnormal. Normal tool wear is primarily caused by friction, high temperatures, and vibrations. In CNC machining, the contact between the tool and metal generates friction, leading to high temperatures and vibrations under complex working conditions. Gradual tool wear occurs during the machining process, which affects tool performance and lifespan. Abnormal wear is caused by various sudden tool failures, which are primarily caused by impact forces generated during milling processes [29]. Tool failure manifests primarily as chipping, cracking, delamination, or plastic deformation.

**Figure 7:**
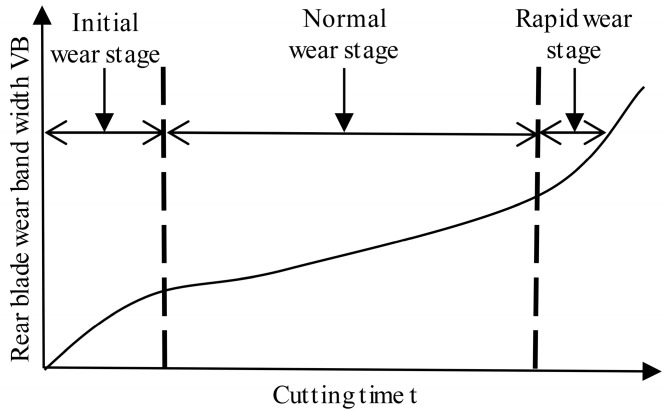
Typical tool wear curve.

Figure 7 illustrates a typical tool wear curve, which indicates that tool wear evolves with increasing cutting time in three main stages: the initial wear, normal wear, and rapid wear stages [30].

**Figure 8:**
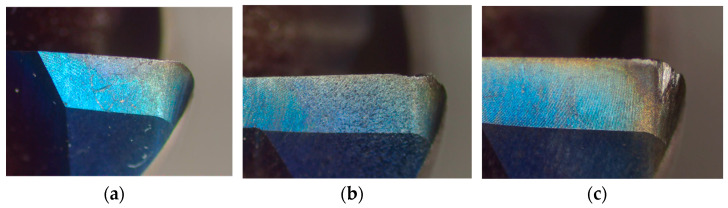
Wear status of milling cutters in different stages: (**a**) initial wear stage; (**b**) normal wear stage; (**c**) rapid wear stage.

The characteristics of the tools vary across different wear stages, as shown in Figure 8, which illustrates the three tool wear stages:

(a) Initial wear stage. Figure 8a shows an image of a tool in the initial wear stage. During this stage, the tool exhibits minor wear patterns as it engages with the workpiece. The initial wear is characterized by a slight removal of material from the tool’s surface.
(b) Normal wear stage. After machining operations, the tool progresses to the normal wear stage, as depicted in Figure 8b. In this stage, the wear pattern becomes more pronounced, reflecting a consistent removal of material from the tool’s surface as the machining operations continue. Although the tool experiences wear, it remains functional.
(c) Rapid wear stage. Figure 8c displays an image of the tool in the rapid wear stage, in which the tool undergoes significant wear, signaling that the end of its lifespan is near. At this stage, the tool exhibits severe damage, such as chipping, cracking, or plastic deformation, indicating imminent failure.

### 3.2. Experimental Design and Data Collection

**Figure 9:**
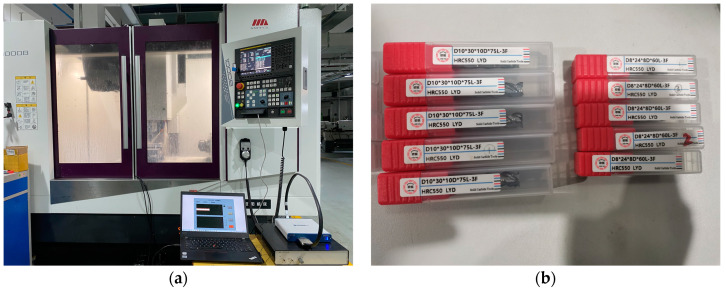
Experimental setup and tool selection: (**a**) CNC machine tool experimental platform; (**b**) HRC550 LYD hard alloy knife.

To obtain raw data for the development of data functions and construction of the algorithms described in the subsequent sections, milling experiments on heat-resistant stainless steel were designed and conducted using an intelligent monitoring system for cutting processes. The workpiece material chosen for acquiring multisource physical data during machining was heat-resistant stainless steel (1Cr11Ni2W2MoV). To collect the data, cutting experiments were conducted on a VMC-1000B vertical machining center. The workpiece was wire-cut into a rectangular block measuring 200 mm × 100 mm × 30 mm to facilitate clamping. Vibration data were collected using an NI acquisition box, filtering amplifier, and RS485 temperature and vibration sensor, as shown in Figure 9a. The cutting tools that were used were HRC550 LYD-type hard alloy end mills, including D8, D10, D12, and D16 double-edge end mills, as shown in Figure 9b.

The milling process involved face milling with a cutter path length of 200 mm and cutting width of 75% the tool diameter. In the face milling experiments, the cutting data were obtained under different conditions and the tool was worn to the stage required for the machining experiments.

**Figure 10:**
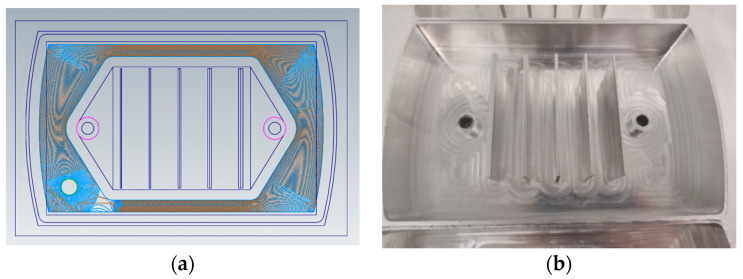
Experimental processing results: (**a**) machining path; (**b**) experimental machining results.

**Figure 11:**
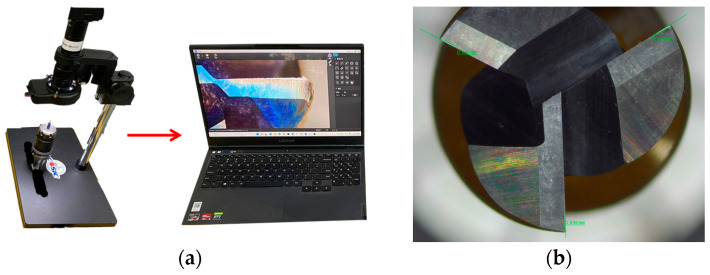
Measurement of tool wear: (**a**) microscopic observation; (**b**) tool wear measurement.

**Figure 12:**
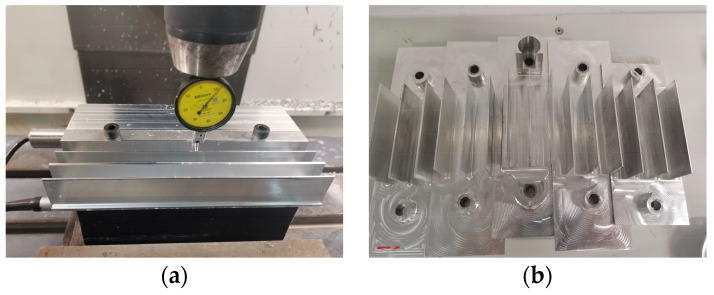
All machined parts processed in the experiment: (**a**) measurement with a dial gauge micrometer; (**b**) experimental processing of thin-walled specimens.

To develop a predictive model of tool wear applicable to various conditions, milling experiments were conducted by varying the cutting parameters. Signals such as the cutting force and vibration acceleration were collected for different sets of cutting parameters and tool wear stages (initial wear, normal wear, and rapid wear). The machining path and structure of the finished parts are shown in Figure 10a and Figure 10b, respectively. In total, 105 milling experiments were conducted using different cutting parameters. After each cutting operation, the tool wear was measured using an HY-H2100 portable electronic microscope, as shown in Figure 11. This allowed the tool wear to be measured after each cutting operation. After machining, each part was examined using a micrometer, as shown in Figure 12a. To efficiently gather additional data, targeted supplementary experiments on thin-walled specimens were designed and conducted, as shown in Figure 12b.

**Figure 13:**
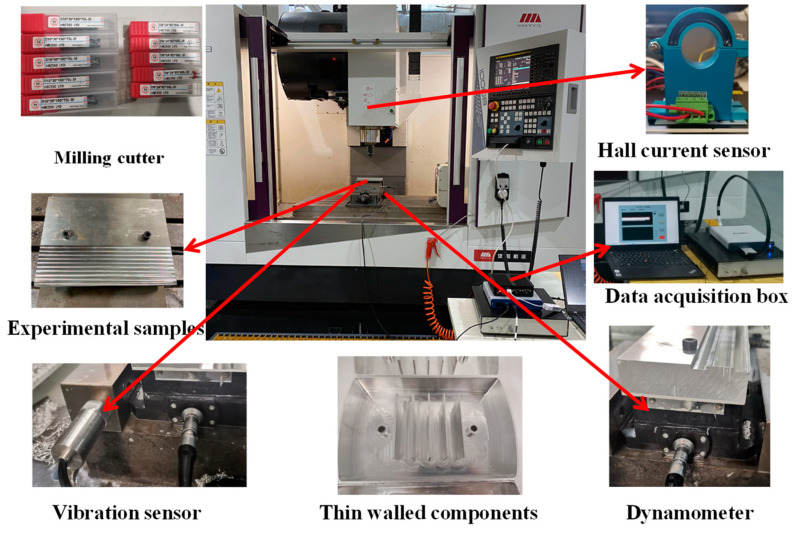
Diagram of the installation positions of the sensors used in the experiment.

Figure 13 shows the sensors used during the experimental machining process and their installation positions, including the arrangement of each sensor, the types of tools, and the clamping of the machining material. The experiment was designed using the aforementioned equipment to prepare for the subsequent data collection, experimental analysis, and derivation of results.

#### 3.2.1. Selection of Experimental Data

To ensure the completeness of the experimental data, each operating condition was treated as a separate experimental objective. Complete thin-wall milling was performed to collect the data and validate the results. The five best thin-walled pieces produced during the experiment were selected for analysis. For each thin-walled piece, 20 datasets were chosen based on the processing parameters. Thus, a total of 100 sets of experimental data were analyzed. The selection of the data focused on the *x*-axis owing to the intense spindle vibration that occurred when the tool was being machined. The experimental parameters and machining conditions are listed in Table 2.

#### 3.2.2. Feature Signal Analysis

**Figure 14:**
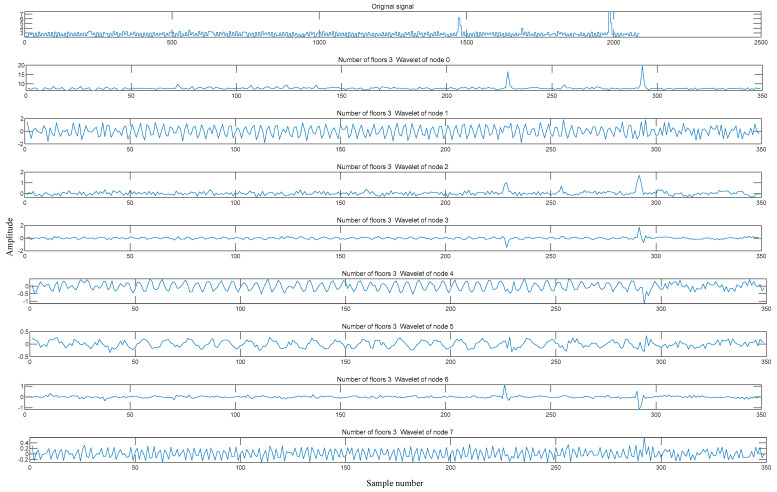
Vibration signals obtained from the three-level wavelet packet decomposition.

**Figure 15:**
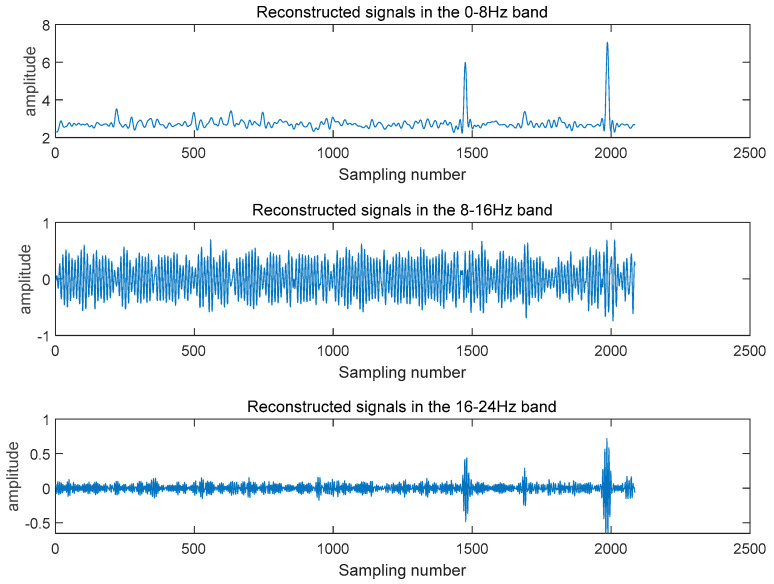
Frequency-domain signal reconstructed by the three-layer wavelet packet decomposition.

**Figure 16:**
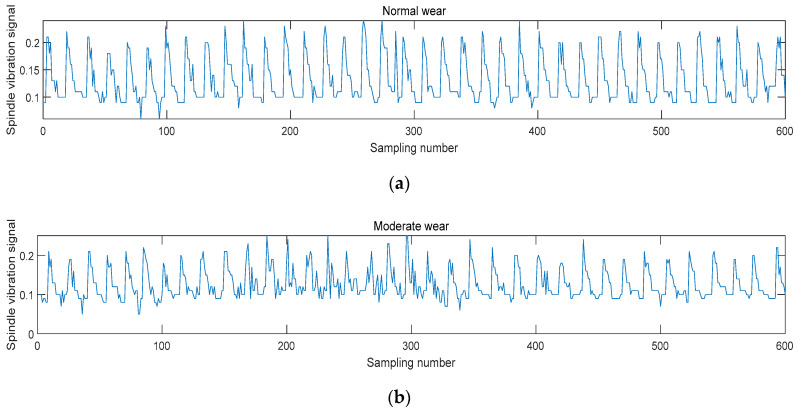

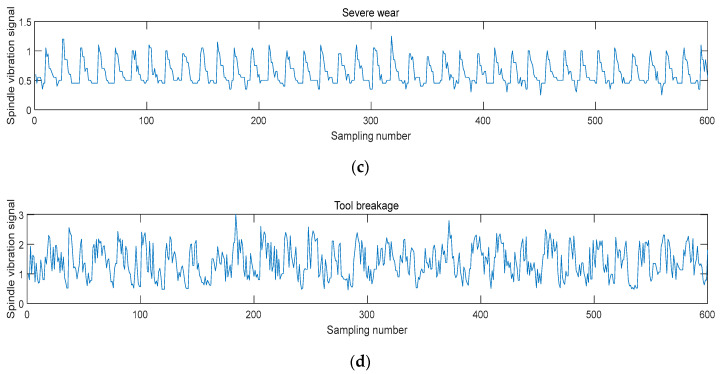
Wavelet analysis of the characteristics of the spindle vibration signals for four different states: (**a**) normal wear cutting state; (**b**) moderate wear cutting state; (**c**) rapid wear cutting state; (**d**) tool breakage cutting state.

Studies on the technology used to monitor machine tool spindle vibrations is crucial for reducing downtime and ensuring product quality. Effective monitoring and diagnostic techniques are often required to monitor the status of equipment. Among the various signals that reflect machine tool status, vibration signals can directly indicate the machining status and dynamic characteristics of a machine tool. Therefore, they are widely used to monitor and identify a machine tool’s status. Taking the collected vibration signal as an example, the vibration signal after the three-level wavelet packet decomposition is shown in Figure 14, and the frequency-domain signals reconstructed after the three-level wavelet packet decomposition are illustrated in Figure 15. Figure 16 shows the spindle vibration signals and their frequency spectra for four different states.

Directly observing the working status of the machine tool spindle from the sensor feature data alone is challenging. Therefore, it is necessary to extract feature coefficients that can effectively characterize the overall spindle and feature parameters that represent the working state under different conditions. These feature parameters can be obtained by analyzing the vibration signal amplitudes, frequencies, and phases. By comparing the feature parameters corresponding to different conditions, the trends in the machine tool spindle vibrations can be determined, which enables abnormal states or faults to be identified. The timely monitoring and diagnosis of the machine tool spindle vibrations can prevent potential failures and enable appropriate maintenance and repair measures to be taken, thus minimizing downtime and maximizing product quality. This process provides critical information for identifying the vibration status and enables a deeper understanding of the operational status of the machine tool.

As shown in Figure 16, the *x*-axis represents the number of points that are sampled and the *y*-axis represents the amplitude of the vibration signal. Figure 16a shows that during normal stable cutting, the changes in the vibration signal are relatively smooth and regular. This occurs because, during normal wear, the wear intensity of the tool edge is uniform, resulting in a stable signal.

The vibration signals exhibited during moderate wear are shown in Figure 16b. Compared to normal wear, very few transient impacts and abrupt high-frequency components are present. When the wear becomes severe, the temporal signal changes become more pronounced. In the rapid wear signal, a large number of nonstationary random components and abrupt frequency components are present, as shown in Figure 16c. Finally, Figure 16d indicates that the signal changes dramatically when the tool reaches the chipped edge stage. The energy of the chipped edge signal reaches its maximum, which produces transient impact components with much greater intensities than the wear signal.

## 4. Results, Discussion, and Analysis

The data collected in the experiments described in the previous section were used to train the model. During the experiment, data were collected from vibration, cutting force, and current sensors on the CNC milling machine worktable in the X-, Y-, and Z-directions. This diverse dataset provided an accurate and comprehensive basis for monitoring tool wear.

### 4.1. LSTM-Based Tool Wear Prediction Model

First, feature extraction was performed on the data collected from the sensors, followed by feature selection. The selected feature vectors were then fed into the LSTM prediction model, and the actual tool wear that occurred during machining served as the training set for the model.

LSTM neural network models possess strong self-learning capabilities for handling sequential data. They possess both long- and short-term memories that enable them to extract deep features from sequential data. This implies that LSTM networks can predict and classify sequential data by learning the patterns and rules within the data. In this section, the preprocessed signal data are used as input to directly train the LSTM model and validate its self-learning capabilities.

The specific steps of Experiment 1 were as follows. First, feature vectors were obtained from the preprocessed normalized signals of the tools, and they served as input for training the model. This approach effectively connected the tool wear with the features of the monitored signals. During the training phase, the collected wear data were used as labels for supervised model training. In the testing phase, the preprocessed signals were used as the test set to validate the LSTM model’s predictions. After approximately 120 iterations, the results showed that the overall change in the loss function stabilized, yielding an RMSE of 0.0281. This indicated that the model performed well in predicting tool wear.

**Figure 17:**
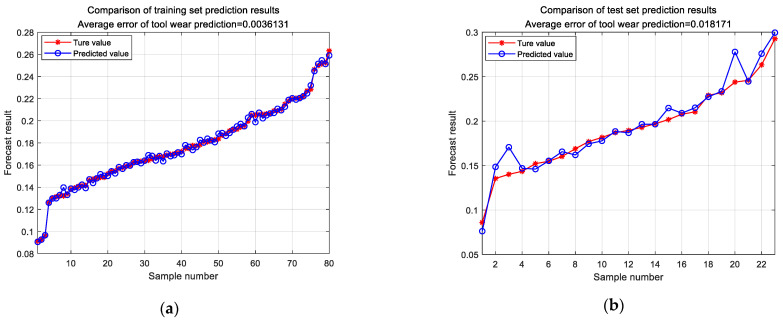
Wear prediction results: (**a**) training set wear prediction; (**b**) test set wear prediction.

Subsequently, the preprocessed monitoring signal data were used to test the model, and the LSTM model was employed for tool wear prediction. The evaluation of the prediction results for the training and test sets is shown in Figure 17. The average MAE of the tool wear prediction was 0.0036 mm for the training set and 0.0181 mm for the test set. These results demonstrated the accuracy and feasibility of the proposed method.

### 4.2. ResNet-Based Tool Wear Prediction Model

**Figure 18:**
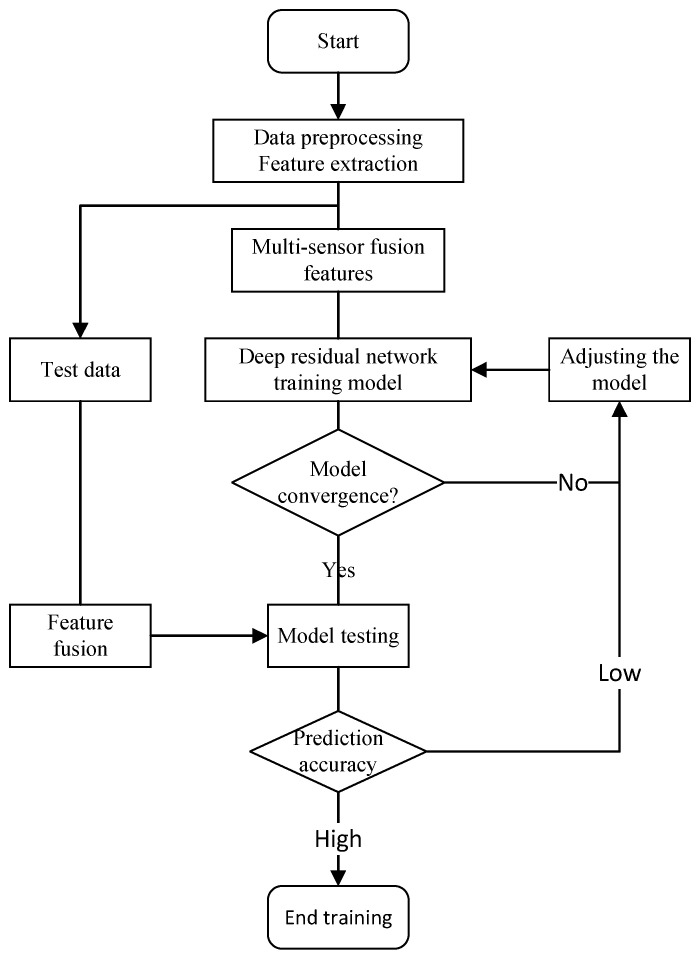
Training flowchart for the ResNet model.

The model training process is illustrated in Figure 18. A fusion feature matrix combining the vibration, current, and cutting force signals was constructed, and this matrix was used to train the tool wear prediction model.

The specific steps of Experiment 2 were as follows. First, feature vectors were obtained from the tool’s full-life monitoring signals, and they served as input for training the model. This approach effectively connected the tool wear with the features of the monitored signals. The experiment indicated that, although the convergence speed of the loss function was relatively slow for the same number of iterations, the overall change in the loss function was significantly smaller, resulting in an RMSE of 0.0182. This indicated that the model performed well in predicting tool wear.

**Figure 19:**
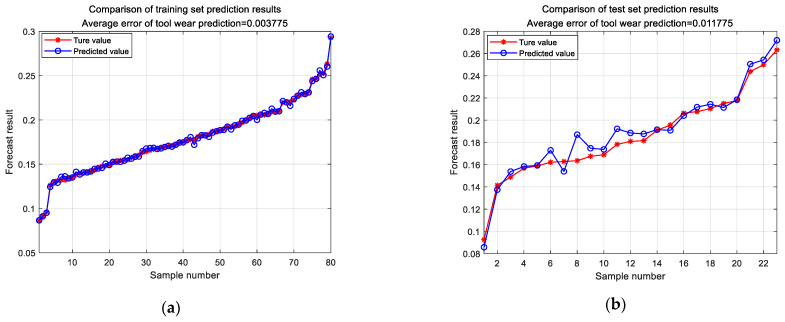
Tool wear prediction results: (**a**) training set wear prediction; (**b**) test set wear prediction.

To further extract features from the monitoring signals, a wavelet packet transform was applied. This method allowed a more refined feature extraction, which improved the accuracy of the tool wear prediction. The feature vectors obtained were used as inputs for the ResNet model. To predict the tool wear, the ResNet model was used, yielding satisfactory results. The evaluation of the prediction results for the training and test sets is shown in Figure 19. The average MAE of the tool wear prediction was 0.0037 mm for the training set and 0.0117 mm for the test set. These results demonstrated the accuracy and feasibility of the proposed method.

Therefore, the results indicated that using feature vectors and the ResNet model for tool wear prediction was effective. Hence, this approach is not only capable of improving the prediction accuracy, but also of contributing to the timely replacement of worn tools during the manufacturing process, thereby enhancing the production efficiency and product quality.

### 4.3. Prediction Model of Tool Wear Based on ResNet-LSTM

**Figure 20:**
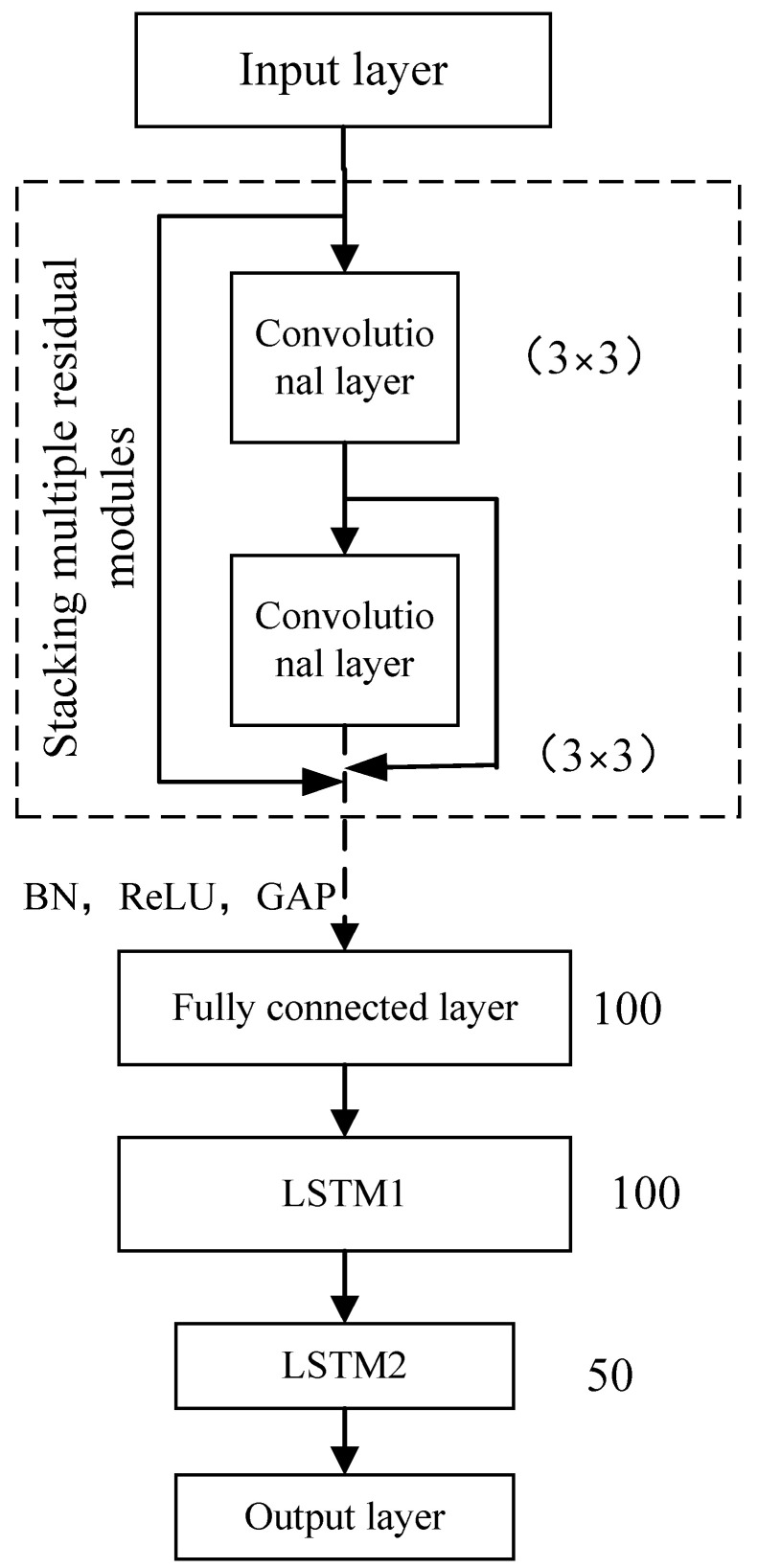
ResNet-LSTM network model.

The ResNet-LSTM network model is illustrated in Figure 20.

The feature signals, which were preprocessed but not denoised, were converted into grayscale images. Two 3 × 3 convolutional layers were used. The convolutional layer of each residual module was defined as a 2 × 2 pooling layer to achieve maximum pooling. The number of neurons was set to 100, and they were connected to the LSTM layer through the pooling layer. Two LSTM layers were set up with a number of hidden layer neurons, as shown in Figure 20. The fully connected layer had one neuron, and its output value represented the predicted tool wear.

Because of the small input dimensions of the ResNet-LSTM network model, the training speed was relatively slow. To improve the training speed, the network model was initialized using the rectified linear unit (ReLU) activation function and the network input dimensions were set to 70 × 70. The batch size of the model was set to 30.

The input parameter of the ResNet-LSTM network model is the preprocessed signal. The number of iterations was set to 500, and the other training parameters were the same as previously described. The tool life data collected by the sensors during the machining process were used as the training set. The differentiation between the training and test sets was the same as that described in previous sections. After training the model, the loss function approached zero and remained stable. The loss function of the validation set had an RMSE of 0.0101. Therefore, the experimental results indicated that the model achieved the expected convergence after approximately 100 iterations.

**Figure 21:**
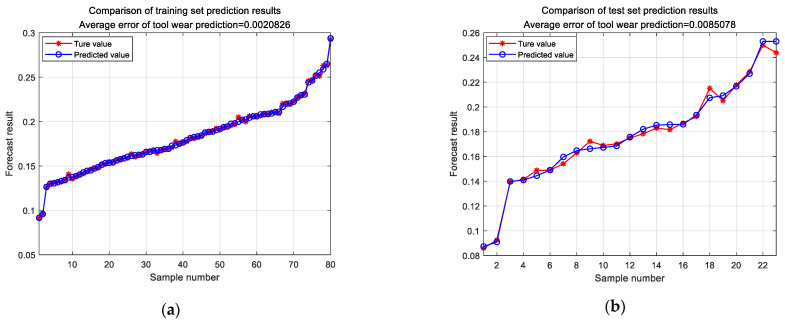
Tool wear prediction results: (**a**) training set wear prediction; (**b**) test set wear prediction.

Subsequently, the tool wear data were tested using the test set, and the predicted results are shown in Figure 21. The average error of the tool wear prediction for the training set was MAE = 0.0021 mm and that for the test set was MAE = 0.0085 mm. These results indicated that this model provided the most accurate prediction, and that the experimental results were consistent with the expected ideal outcomes.

Table 3 compares the prediction accuracy and wear error of the three network models using the same tool data as the test set. By comparing the prediction results of each model, the following conclusions can be drawn. When using the ResNet network model, wear prediction was performed by extracting the feature vectors of the signal. The experimental results showed that as the number of model layers increased, the loss function significantly decreased. Moreover, as the network depth increased further, the accuracy approached saturation without decreasing. However, after adding two LSTM layers, the accuracy further improved, indicating that the feature extraction of the LSTM model was more effective, improving the tool wear prediction. Finally, the ResNet-LSTM model was proposed by combining residual neural networks with the LSTM network model, which significantly improved the prediction accuracy of the model compared to the individual LSTM and ResNet models. The ResNet-LSTM model yielded an average prediction error of 0.0085 mm and a tool wear prediction accuracy of 98.25%.

## 5. Conclusions

With the widespread application of CNC machine tools, the accurate monitoring of machining process states and the precise identification of tool wear have become increasingly important. Experiments on tool wear prediction during machine tool processing were designed, and a tool wear prediction system based on multi-sensor information fusion was proposed. The main conclusions of this study are as follows:

(1) The use of the Kalman filtering algorithm for feature extraction and the fusion of multi-sensor signals provided a basis for subsequent model training.
(2) Using the LSTM network model and training it with the fused features of three signals generated a favorable prediction performance, although the signal features were not distinct.
(3) The ResNet model was constructed for experiments with the same tool wear data, resulting in improved accuracy but a slower convergence speed for the loss function.
(4) The ResNet-LSTM model was constructed by combining residual neural networks with the LSTM network model, which significantly improved the prediction accuracy compared to the individual LSTM and ResNet models. Moreover, the combination of residual neural networks and LSTM networks exhibited a certain adaptive denoising capability at the front end of the network for feature extraction, thereby enhancing the signal feature extraction capability.
(5) Finally, the reliability of the method was verified through actual machining experiments.

However, in actual production and machining processes, more complex machining phenomena, in which the machining efficiency involves multiple influencing factors, are often encountered. This study collected and processed data from only four working conditions. Therefore, in future research, we will aim for a more comprehensive understanding of the tool wear status that occurs during machining and conduct more in-depth experiments and data analyses of the complex working conditions encountered during machine tool processing. In addition, high temperatures significantly affect tool life, but the influence of high temperatures on tool life was not considered in this study because of the use of cutting fluids. Accordingly, in future work, we will consider adding external temperature sensors to monitor the impact of high temperatures on tool life.

## Figures and Tables

**Table 1:** Initialization parameters for the LSTM network model.

Parameter Definition	Parameter Settings
Optimization method	Adam
Network input dimension	3 × 40
Loss function	RMSE
Batch size	20
Dropout	0.5
Initial learning rate	0.1
Epoch	200

**Table 2:** Experimental parameters and machining conditions in the milling process.

Number	Spindle Speed (r/min)	Feed (mm/min)	Cutting Depth (mm)	Tool Wear Status	Remarks
1	3000	400	0.2	Normal wear stage	Normal
2	3000	400	0.3		
3	3000	400	0.4		
4	3000	400	0.5		
5	3000	400	0.6		
6	3000	500	0.2	Moderate wear stage	Normal
7	3000	500	0.3		
8	3000	500	0.4		
9	3000	500	0.5		
10	3000	500	0.6		
11	3000	600	0.2	Rapid wear stage	Abnormal
12	3000	600	0.3		
13	3000	600	0.4		
14	3000	600	0.5		
15	3000	600	0.6		
16	3000	800	0.2	Tool breakage stage	Abnormal
17	3000	800	0.3		
18	3000	800	0.4		
19	3000	800	0.5		
20	3000	800	0.6		

**Table 3:** Comparison of tool wear prediction results.

Model	MAE (mm)	RMSE (mm)	$R^{2}$
LSTM	0.0182	0.0281	0.8744
Resnet	0.0118	0.0182	0.9745
ResNet-LSTM	0.0085	0.0101	0.9825

## Data Availability

Data are contained within the article.
